# Signal and Contrast Optimization With Predicted Excitations (SCOPE) for Accelerating Large FOV Body Imaging at UHF

**DOI:** 10.1002/mrm.70362

**Published:** 2026-03-26

**Authors:** Tobey D. Haluptzok, Simon Schmidt, Gregory J. Metzger

**Affiliations:** ^1^ Center for Magnetic Resonance Research (CMRR) University of Minnesota Minneapolis Minnesota USA; ^2^ Medical Physics in Radiology German Cancer Research Center (DKFZ) Heidelberg Germany

**Keywords:** 7 Tesla, AMORE, body imaging, fast spin echo, FSE, TIAMO, TSE, turbo spin echo, UHF MRI

## Abstract

**Purpose:**

Large FOV turbo‐spin‐echo (TSE) imaging at ultra‐high field (UHF) remains challenging due to B_1_^+^ inhomogeneity and peak specific absorption rate (pSAR) limitations. This work presents a new time‐interleaved acquisition of modes (TIAMO) framework called SCOPE (Signal and Contrast Optimization with Predicted Excitations), which overcomes these challenges by enabling reduced repetition time (TR) without compromising image quality.

**Methods:**

TIAMO is implemented to operate at half the TR of standard static‐shim acquisitions, maintaining scan time parity with traditional acquisitions. To mitigate the expected contrast and signal loss from TR reduction, we developed a novel optimization framework that finds spatially exclusive RF modes, ensuring each voxel is predominantly excited by a single mode, thereby restoring the effective TR. To find these modes, the optimization utilizes an extended phase graph (EPG) model formulated to simulate the TSE signal with interleaved RF excitations. This new signal model is incorporated into the SCOPE framework to compute subject‐specific optimal shims. Previous TIAMO methods are reformulated using this new signal model to elucidate performance differences.

**Results:**

SCOPE produced more homogeneous images with improved pSAR efficiency by alternating SAR hotspots between modes. In vivo measurements in the prostate and kidneys strongly correlated to signal prediction and demonstrated superior image quality compared to prior TIAMO methods. The new signal model clarified performance tradeoffs between TIAMO strategies.

**Conclusion:**

SCOPE enables rapid, contrast‐preserving TSE TIAMO imaging with reduced TR, addressing the longstanding scan‐time penalty of TIAMO. This work establishes a foundation for real‐time, model‐driven pTx optimization in large‐FOV UHF imaging.

## 1 Introduction

Ultra‐high field (UHF) MRI (≥ 7 T) offers improved signal‐to‐noise ratio (SNR) and enhanced tissue contrast [1, 2, 3, 4, 5, 6] as compared to lower field strengths but also introduces significant challenges due to the shortened in vivo wavelength (∼11 cm at 7 T). This shortened wavelength leads to spatially varying electromagnetic (EM) fields, which in turn cause B_1_^+^ inhomogeneity and increased peak specific absorption rate (pSAR). While these effects are relatively minor in smaller regions like the head or knee, they become prohibitive in larger anatomical targets such as the abdomen and pelvis, resulting in signal dropout, spatially inconsistent contrast, and potential safety concerns; all of which hinder clinical translation.

Parallel transmission (pTx) methods have been developed to mitigate these challenges, with various techniques extensively reviewed in the literature [7, 8, 9, 10, 11, 12, 13, 14]. The simplest and most widely utilized form of pTx is static RF shimming, where the phase and magnitude of each transmit channel are optimized to balance transmit efficiency, homogeneity, and pSAR constraints [10, 11]. Although static shimming improves performance within moderate‐sized FOVs, it is often insufficient for large FOV imaging, either due to residual B_1_^+^ inhomogeneity, power constraints, or pSAR limitations.

To expand spatial coverage and improve homogeneity, the time‐interleaved acquisition of modes (TIAMO) [12, 15] method was introduced. In TIAMO, multiple RF shims, often referred to as modes, are alternated across repetition times, generating two complementary images that are combined using a virtual channel parallel imaging formulation for improved accelerated performance. For gradient echo (GRE) sequences, a least‐squares (LS) optimization of the B_1_^+^ between two modes has been shown to be effective [12]. However, this signal combination model is suboptimal for turbo‐spin‐echo (TSE) imaging, which is highly sensitive to the maximum flip angle (FA) achieved in each voxel rather than the LS combined B_1_^+^ fields. The AMORE method used this intuition to develop a new cost function, requiring at least one of the modes to reach a target FA [16]. AMORE also introduced the concept of mode localization, which enforced high excitation efficiency in specific ROIs like the prostate. Despite these advances, TIAMO methods still suffer from a fundamental tradeoff; increasing the number of phase‐encode lines increases scan time or requires higher parallel imaging acceleration, which reduces SNR. A different method to address B_1_^+^ inhomogeneity is direct signal control (DSC) which modifies RF shims and FAs across the refocusing train and optimizes the different RF shims based on an EPG based signal prediction.

In this work, we integrate the signal prediction ideas from DSC into a TIAMO optimization pipeline by developing a new two mode TSE‐TIAMO extended phase graph (EPG) signal model. We show that the signal model predictions are correlated with in vivo measurements and show that reducing TR has minimal impact on signal and contrast, so long as the two TIAMO modes are spatially exclusive, which matches intuition as the effective TR experienced by the spins is twice that which is prescribed on the scanner when the modes are perfectly exclusive. We then leverage this model in a real‐time, dictionary based, Signal and Contrast Optimization via Predicted Excitations (SCOPE) pipeline, which solves for optimal transmit modes that preserve contrast, improve homogeneity, and reduce pSAR on the order of a few seconds.

## 2 Methods

### 2.1 Simulating the TSE‐TIAMO Signal

To model the TSE signal resulting from interleaved RF excitations in TIAMO acquisitions, we developed a modified EPG [17, 18, 19] model that incorporates mode‐specific FAs. In this model, we aim to predict the signal of each individual voxel by looking at the FA achieved in that voxel by each mode. Since on‐the‐fly EPG simulations can be computationally intensive, we build up a signal dictionary by varying the FA of the two prescribed modes and utilize this dictionary later during real‐time optimization. The flip angles simulated are based on the excitation flip angle, with the first refocusing pulse being twice the excitation FA and each successive refocusing FA being 14/9 times the excitation FA. For example, a 90° excitation would have a 180° first refocusing FA and then 140° for all subsequent refocusing FAs. This FA schedule was chosen as it is a common setting used for TSE scans. The two flip angles are simulated in an interleaved fashion, each having an echo train length (ETL) of 9, a TE defined as the time when the fifth echo occurs, a signal defined as the F0 state at the fifth echo, and a TR defined as the time between excitation pulses. The two modes are coupled together by keeping track of the longitudinal magnetization available at each successive excitation. This is done by calculating a $1-e^{-t/T1}$ longitudinal recovery after each refocusing train accounting for T_1_, TR, and flip angles from the previous mode.

To cover most imaging situations, these simulations are performed for multiple flip angles, TEs and TRs, and different T_1_ and T_2_ relaxation constants. Working off the concept of localization presented in AMORE, we define the highest FA mode on a voxel‐wise basis as the major mode and the lower FA mode to be the minor mode. The major mode excitation FA varied from 0° to 180° in steps of 9° and the minor mode FA was simulated as a relative power calibration with respect to the major mode from 0% to 100% in steps of 5%. TRs ranged from 1000 to 6000 ms in steps of 1000 ms. TEs ranged from 40 to 110 ms in steps of 10 ms. T_1_ relaxation values ranged from 1000 to 2000 ms in steps of 20 ms, and T_2_ values ranged from 50 to 300 ms in steps of 5 ms. The result of sweeping these scan parameters was then saved as a 6D array which is used for real‐time optimization described in the following section. Code used to generate this EPG dictionary and other downstream optimizations is available at https://github.com/Tobey‐Haluptzok/SCOPE_TIAMO. Simulated EM field data can be found at https://doi.org/10.13020/zddj‐z486.

### 2.2 SCOPE Framework

**FIGURE 1:**
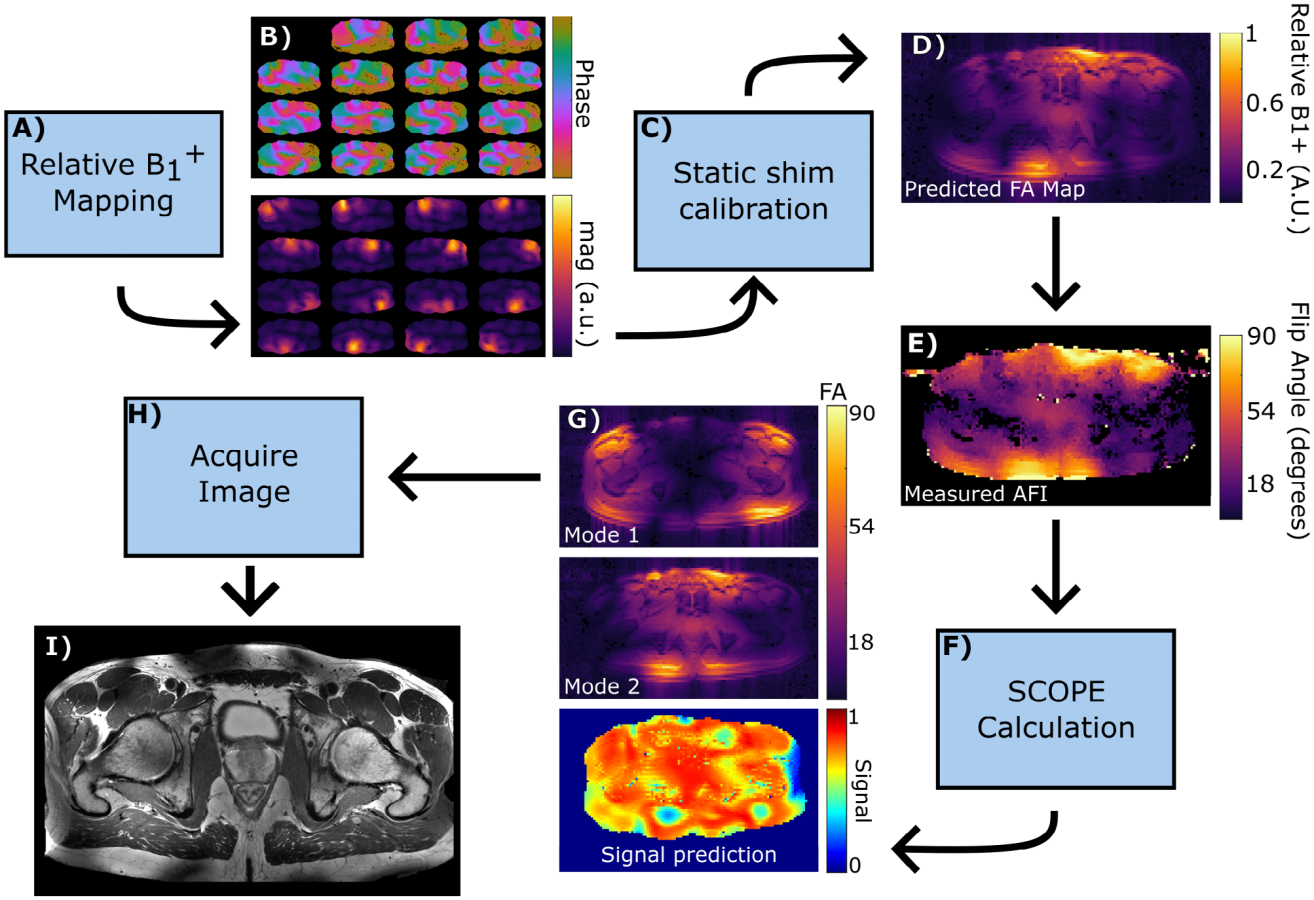
A flowchart depicting the workflow used to acquire SCOPE images. The first step (A) is a standard B1 relative acquisition which is reconstructed into channel‐wise magnitude and phase maps (B). Some target ROI, in this case the prostate, is then defined and an efficiency static shim is calculated (C) which predicts a relative B_1_^+^ pattern (D) which is then used to power calibrate using AFI maps (E). Now that the system is power calibrated relative to the efficiency shim, we perform a real‐time SCOPE optimization (F) which finds the two B_1_^+^ modes and predicts a contrast and/or signal map (G). The two modes are interleaved in a TSE acquisition (H) which is reconstructed using virtual channels and GRAPPA [20] to get the final image in (I).

The SCOPE framework computes optimal RF shimming solutions using the TSE signal predicted in the EPG model described above and solves the multi‐objective optimization problem in Equation (1). A flowchart depicting calculations and intermediate results of the SCOPE method is shown in Figure 1.

To optimize RF shims, the framework predicts the signal corresponding to FA distributions from the two modes to be used during acquisition. The FA distribution for each mode is calculated by applying a complex shim vector to channel‐wise B_1_^+^ maps. In the case of the two‐mode TIAMO explored in this work, the result is a N_v_ × 2 array where N_v_ is the number of voxels. The EPG dictionary is then used to predict the signal for each voxel, resulting in an N_v_ × 1 signal vector, **S**.

This signal prediction step is implemented in three different ways, with each method requiring different amounts of information about the imaging sample. Since relaxation properties are not necessarily known a priori and may vary across the imaging volume, a general signal prediction method that is independent of T_1_ and T_2_ is needed. To address this, we perform a singular value decomposition (SVD) of the dictionary at each fixed TE and TR to decouple the FA signal dependence from relaxation signal dependence. This SVD analysis is performed by pulling a 4D array out from the EPG dictionary corresponding to a given TE and TR. The array is then reshaped into a 2D matrix with dimensions [N_major_ × N_minor_, N_T1_ × N_T2_], where N is the number of steps in the respective parameter, thereby coupling the B_1_^+^ variables into the first dimension and contrast variables into the second dimension. This array is then decomposed into its major components using an SVD, where the first left singular vector represents the B_1_^+^ dependent signal behavior and the right singular vector the relaxation parameter signal dependence. Reshaping the B_1_^+^ singular vector into a 2D array of size N_major_ × N_minor_ creates a signal prediction map which is used to map the two FA at each voxel into a predicted signal. A second way to utilize the EPG dictionary is when a single T_1_/T_2_ pair of relaxation properties is known. For this method, the exact 2D B1+ signal dependence is extracted from the dictionary at a prescribed TE, TR, T_1_, and T_2_. The third way to utilize the EPG dictionary is when two T_1_/T_2_ combinations are known. For example, if one is interested in maximum contrast between two different tissue types this method is used to create a *contrast* prediction map by subtracting the two associated signal prediction maps. Throughout this work, we refer to all of these as “signal predictions,” with the understanding that the third EPG lookup method targets contrast specifically.

Once a predicted signal vector is computed for a given ROI, scalar metrics are defined to guide optimization. A natural starting point is to maximize the mean signal while remaining within pSAR safety limits. Although pSAR could be enforced as a hard constraint, in this work we incorporate it as a weighted penalty term, which is mathematically equivalent to a constrained optimization via Lagrange duality, and calculate it using virtual observation points (VOPs) [21, 22, 23]. However, optimizing only for mean signal and pSAR may lead to highly localized excitation patterns that achieve the highest signal in only a limited subset of the ROI, while leaving other regions with low flip angles and low signal. Such solutions, while technically efficient, are undesirable in clinical imaging where consistent signal across the anatomy is required. To address this, a third metric is introduced to promote spatial homogeneity. In this work, we use the coefficient of variation (CV) of the predicted signal as a normalized measure of uniformity.

The combination of these three metrics yields the final multi‐objective loss function:

(1) $$\min_{x}-\text{mean}\left(S\left(x,B_{1}\right)\right)+\lambda_{1}\sqrt{\text{pSAR}\left(x,\mathrm{VOP}\right)}+\lambda_{2}\cdot \mathrm{CV}\left(S\left(x,B_{1}\right)\right)$$

where **x** denotes the concatenated shim vectors across modes, and *λ*_1_ and *λ*_2_ are weighting parameters that determine the trade‐off between signal strength, pSAR efficiency, and homogeneity.

#### 2.2.1 In Vivo Measurements Compared to the EPG Model

To verify that the EPG model predictions correlate with actual measurements, in vivo experiments were conducted in two healthy volunteers. All experiments were performed under an IRB‐approved protocol on a 7 T whole‐body MRI system (Magnetom 7T, Siemens Healthineers, Erlangen, Germany) using a custom‐built 16‐channel transmit/32‐channel receive shielded loop‐dipole array [24].

Axial TSE scans of the prostate were acquired using a phase‐only RF shim at a TR of 5000 ms and were repeated using TIAMO at a reduced TR of 2500 ms. The TIAMO acquisitions were performed by applying this phase‐only shim as both the major and minor mode with the minor mode fractionally scaled from 0% to 100% in steps of 10%, while the major mode was held constant at a 90° excitation FA, verified by actual flip angle imaging (AFI) [25]. A similar protocol was implemented for renal imaging in the coronal plane for a second volunteer, using a static shim acquisition at TR = 3500 ms and TIAMO acquisitions at TR = 1750 ms. As in the prostate scans, the minor mode was systematically varied while the major mode maintained 90° excitation FA.

The resulting images were analyzed by segmenting high‐signal and low‐signal regions within the anatomies and displaying voxel‐wise histograms to quantify signal distributions. These experimental results were then compared with the signal predictions obtained from the EPG dictionary using the SVD‐based B_1_^+^ signal dependence for the prescribed TE and TR.

#### 2.2.2 Comparing SCOPE to Previous TIAMO Methods via L‐Curves

To contextualize SCOPE in reference to previous methods, we implemented two previously published TIAMO optimization strategies, LS‐TIAMO [12] and AMORE [16], using a signal prediction framework similar to what is used in SCOPE. For each method, corresponding signal prediction maps, shown in Figure S1, were constructed to enable direct comparison under the same signal prediction framework. These maps were then used in a SCOPE‐style optimization to evaluate performance using the same metrics: mean signal, pSAR, and coefficient of variation (CV).

To compare the performance trade‐offs inherent in each method, L‐curve plots were generated by varying the homogeneity weighting parameter λ_2_ in Equation (1), while keeping the *λ*_1_ SAR weight fixed. This analysis was performed on simulation data generated in Sim4Life 8.0 (ZMT Zurich MedTech AG) using a 16‐channel loop‐dipole transceiver array [26] positioned over the kidneys of the Duke human body model (IT'IS Foundation, Zurich, Switzerland). The L‐curve analyses were carried out in two anatomically relevant ROIs: a kidney‐specific ROI and a larger ROI consisting of an entire axial slice intersecting the center of the kidneys. L‐curves were computed at three different TR values (1500, 3000, and 6000 ms) to reflect the variability in signal prediction maps at different TRs. For the kidney‐specific analysis, where relaxation properties have been reported in the literature [27], we used a B_1_^+^ dependent contrast map derived from two tissue species. For the full‐slice analysis, where relaxation properties are more heterogeneous, we used the SVD‐derived B_1_^+^ dependent signal map.

To ensure robust optimization and avoid local minima, four random initializations were used for each value of *λ*_2_.

#### 2.2.3 In Vivo Imaging

To evaluate the performance of SCOPE‐optimized shims in practical imaging scenarios, in vivo TSE acquisitions were performed in the kidneys and prostate using a 16Tx/32Rx shielded loop‐dipole array. For kidney imaging, both local and non‐local RF shim solutions were computed using SCOPE and AMORE. In the renal studies, the kidneys were segmented from a low‐resolution GRE scan to define the local ROI, and the remaining body area in each slice was used as the non‐local ROI. The local and non‐local shim optimizations were performed on the same anatomical masks in both methods.

Prostate imaging was performed using AMORE‐based localized shimming and compared against statically shimmed acquisitions. The localized AMORE solution was acquired at TR = 3000 ms and the static shim reference acquisition was performed at TR = 6000 ms.

In all cases, RF shims were computed using relative channel‐wise B_1_^+^ maps [28] and subsequently power‐calibrated using either an AFI sequence or a saturated turbo FLASH acquisition [29, 30]. A summary of imaging parameters for all in vivo acquisitions is provided in Table S1.

## 3 Results

### 3.1 EPG Simulation

**FIGURE 2:**
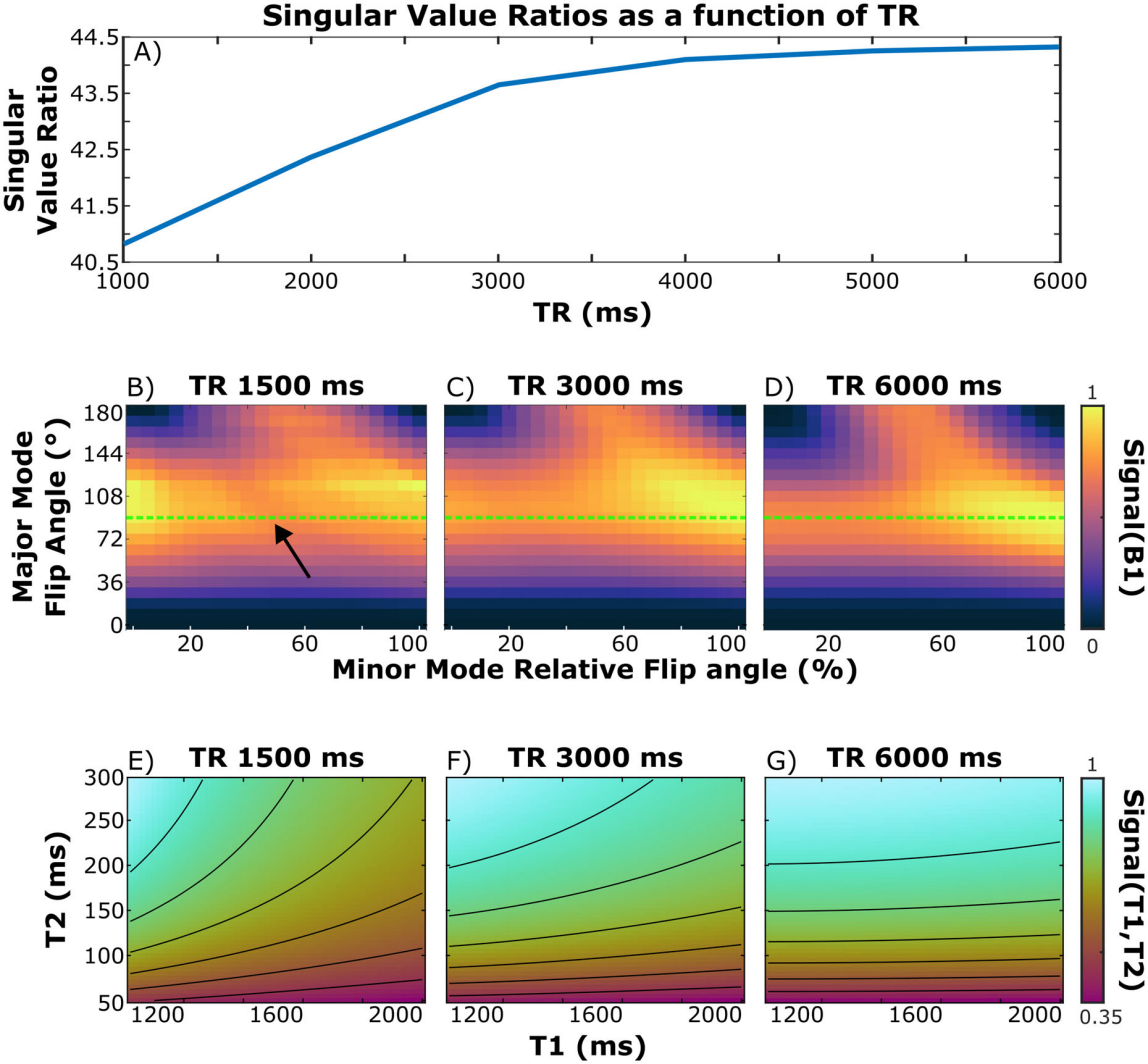
SVD analysis of the EPG dictionary. The largest to second largest singular value ratio as a function of TR is shown in (A). With the largest singular value being at least 40× as large as the second singular value, the dictionary is nearly singular and taking the largest left and right singular vectors will provide a good approximation of the dictionary. Singular vectors are shown at TRs of 1500 ms (left), 3000 ms (middle) and 6000 ms (right). Reshaped left singular vectors create the B_1_^+^ dependent signal maps and reshaped right singular vectors create the contrast dependent signal maps. At the shortest TR of 1500 ms in (B), there is a deeper null in the B_1_^+^ dependent signal map when the major mode achieved a 90° excitation, and the minor mode has a relative calibration between 40% and 60% (black arrow). When looking at the contrast dependent signal maps (E–G), as TR increases, T_1_ dependence in the relaxation dependent signal maps decreases. This is visualized by isocontour lines plotted on the images where a perfect horizontal isocontour line being equivalent to no T_1_ dependence.

**FIGURE 3:**
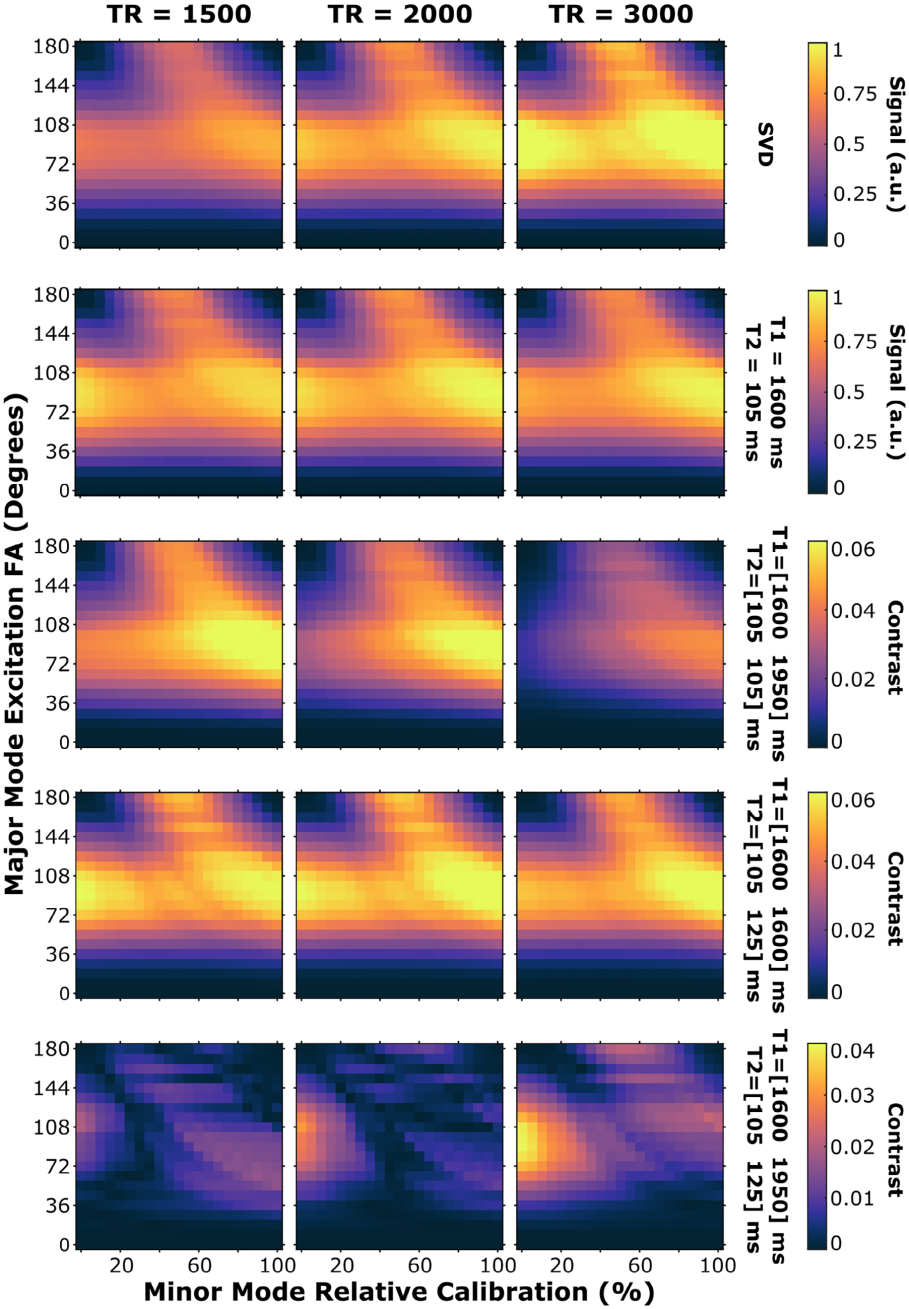
EPG results displaying how the B1 signal maps change with varying TR for input methods 1, 2, and 3. The SVD and single species signal prediction maps in the first two rows show that as long as one of your modes has an excitation FA in the range of 90°, signal will be reasonably high, regardless of the minor mode FA. In the contrast prediction maps where only T1 or only T2 values are different, contrast is also relatively insensitive to minor mode FA. However, when two chemical species have different T1 and T2 values, as shown in the bottom plot, the minor mode FA can have a significant effect on contrast.

Figure 2 shows the ratio of the first to second singular value and demonstrates a more than 40‐fold increase for all TRs evaluated, justifying the use of the low‐rank approximation for signal prediction in SCOPE. Signal prediction maps derived from the left singular vectors show the expected peak signal when the major mode achieves a 90° excitation, consistent with prior AMORE results. At shorter TRs (e.g., 1500 ms), the predicted signal decreases by 10%–20% when the minor mode relative calibration is between 30% and 60% indicating that mode exclusivity, and therefore the update from AMORE to SCOPE for signal prediction becomes increasingly important as TR is reduced. The corresponding relaxation dependent signal maps (from the right singular vectors) show that the signal at long TRs (e.g., 6000 ms) is relatively insensitive to T_1_, while shorter TRs introduce stronger T_1_ dependence, as expected. Figure 3 illustrates how signal prediction maps depend on TR and compares relaxation‐specific images to SVD‐derived images. These signal prediction images show that at lower TRs the signal becomes increasingly sensitive to relative flip angle of the minor mode. Furthermore, we see that the SVD images have a very high resemblance to the signal images for the given T_1_ and T_2_ relaxation values evaluated. To assess the impact of major and minor mode on contrast, Figure 3 also shows B_1_^+^ signal dependent maps when two species have different T_1_s, different T_2_s, and both different T_1_s and T_2_s. T_1_‐only and T_2_‐only contrast images have optima when both modes achieve 90° excitations. However, when two species have different T_1_ and T_2_ relaxation times, with an increased T_2_ accompanying an increased T_1_, as is commonly the case in vivo [27], the two contrasts compete, resulting in an optimum when the minor mode has zero flip angle or B_1_^+^. As the TR is reduced, there is a larger contrast variance with respect to the relative calibration of the minor mode. In the most extreme case, at a TR of 1500 ms, a perfectly calibrated first mode can still result in close to zero contrast if the minor mode is as little as 30% the flip angle of the major mode.

### 3.2 In Vivo EPG Model Comparison

**FIGURE 4:**
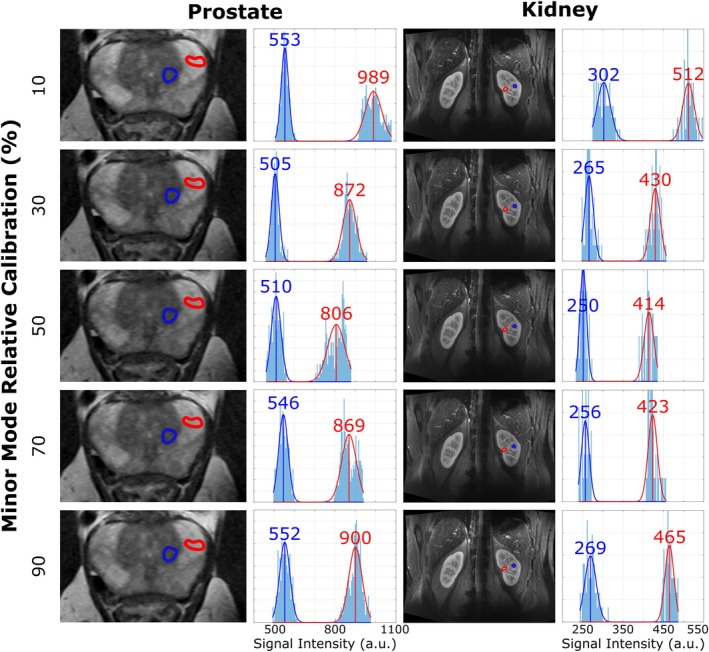
In vivo TSE images of the prostate and kidneys using two modes. While the phases of the two modes were identical and optimized for transmit efficiency in the target organ, the primary mode was power calibrated, and the secondary mode was incremented from 0% to 100% relative calibration in increments of 10%. Signal intensities from two ROIs of varying contrast in each anatomy are shown in voxel‐wise histograms. Since all histograms are on the same x scale for a given anatomy, the shift to the left with minor mode calibration between 30% and 70% represents a lower SNR while the difference between the two ROI histograms on a single plot is the corresponding contrast to noise ratio (CNR). The characteristic signal minima between 30% and 70% minor mode calibration agrees well with the EPG dictionary prediction.

In vivo scans of the prostate and kidneys demonstrated close agreement with EPG model predictions. Figure 4 shows axial and coronal TSE images acquired at reduced TR with varying minor mode relative calibration levels. Signal histograms from segmented high‐ and low‐signal regions confirm that the maximum signal occurs when the minor mode is either fully suppressed or fully calibrated. Intermediate minor mode calibrations between 30% and 70% consistently produced reduced signal in all four segmented regions, which is consistent with the EPG model's predicted signal behavior. These results justify the use of the EPG signal model in SCOPE optimizations.

### 3.3 SCOPE, AMORE, and LS‐TIAMO L‐Curves

**FIGURE 5:**
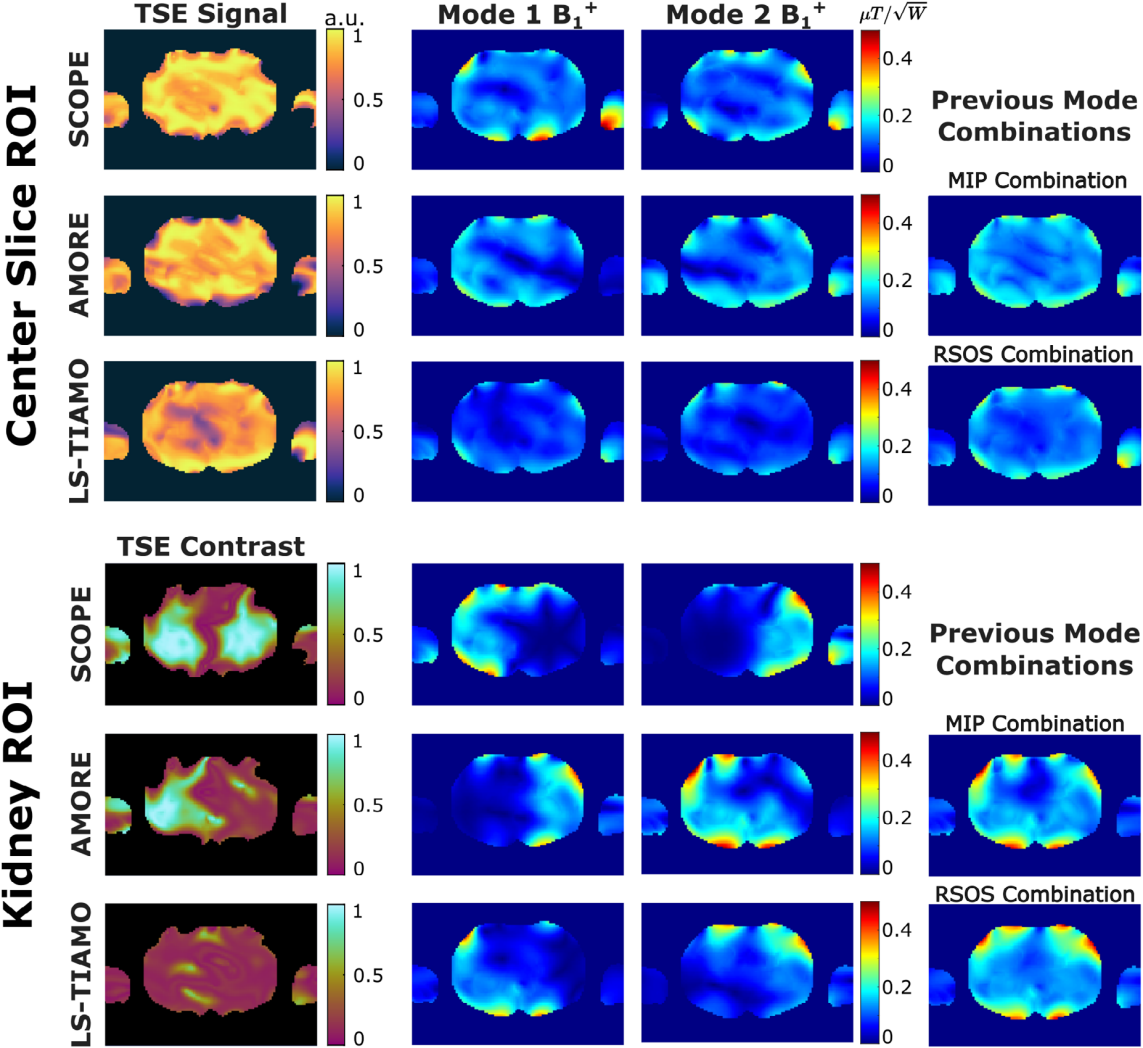
Example maps using SCOPE, AMORE, and LS‐TIAMO optimization methods. The leftmost column is the EPG‐predicted TSE contrast for the kidneys using kidney relaxation parameters from literature and the signal for the central slice using the SVD signal approximation. The two middle columns are B_1_^+^ maps after applying calculated optimal shims, and the rightmost maps are the combination of the two modes using previous methods. The AMORE and LS‐TIAMO optimizations produce reasonable combined field, confirming that the optimizer for these methods found good solutions for the respective loss functions. For both AMORE and LS‐TIAMO, justifying that the optimization found reasonable solutions for both respective loss functions. The signal prediction maps show that both AMORE and LS‐TIAMO would generate signal over the entire volume but not as much signal as SCOPE. The contrast prediction maps show that both LS‐TIMAO and AMORE fail to create uniform contrast over both kidneys at the tested TR of 1750 ms.

**FIGURE 6:**
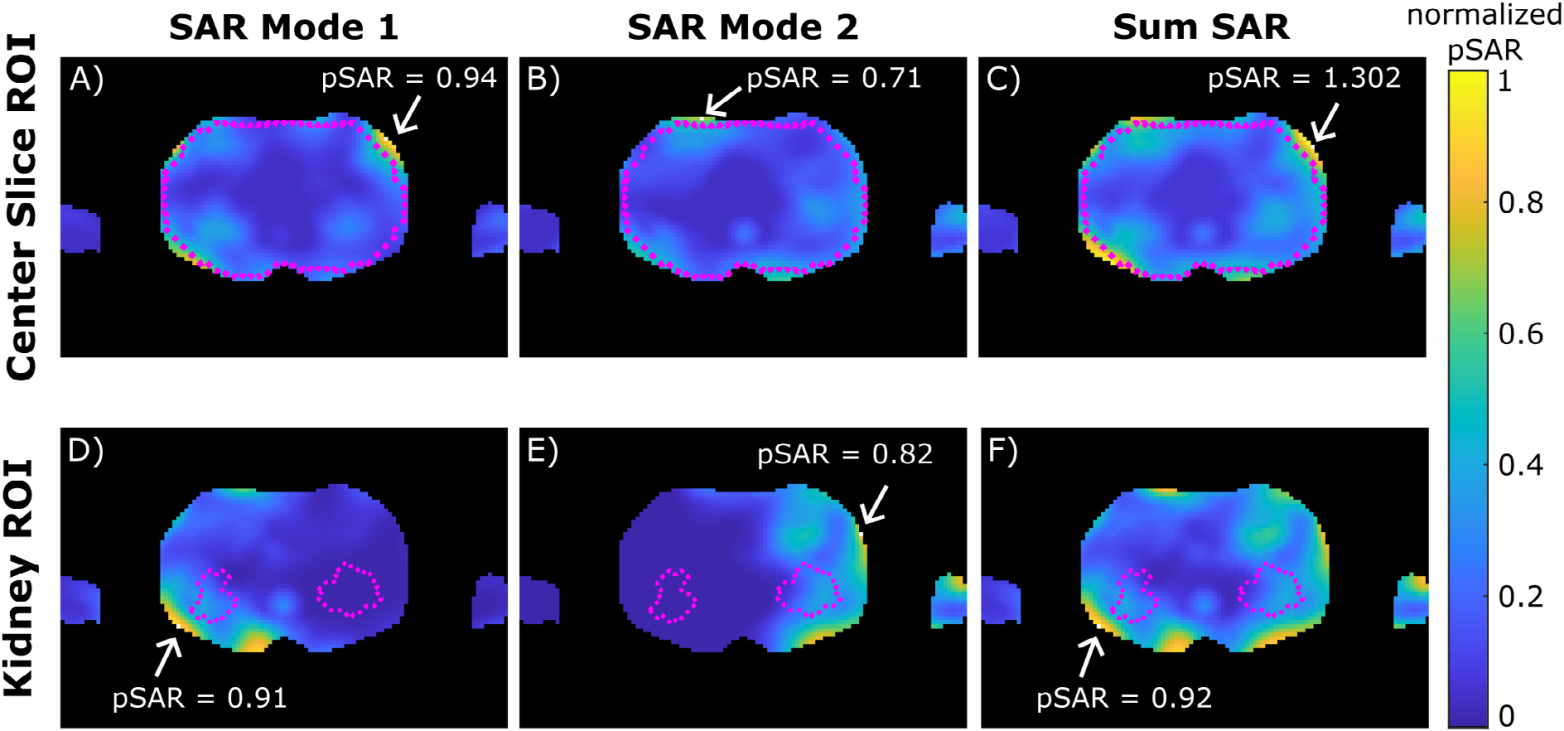
Ten‐gram averaged SAR maps from the shimming solutions shown in Figure 6, for both the center slice ROI and kidney ROIs (outlined in magenta). We see that for both ROIs, each mode has a different pSAR location which minimizes the cumulative worst case pSAR. In particular, when shimming the entire slice, the pSAR increased around 30%. For the kidney ROI, the SAR maps were orthogonal enough to maintain the same cumulative pSAR as the first mode only. The end result is a TIAMO acquisition with half the TR and no SAR penalty.

Predicted TSE signal and contrast maps with corresponding B_1_^+^ distributions for SCOPE, AMORE, and LS‐TIAMO are shown in Figure 5. Each method was tested with a kidney‐specific and full‐slice ROI. In both settings, LS‐TIAMO produced the least homogeneous signal, with persistent low‐signal regions. AMORE showed improved performance when generating signal in the large ROI but resulted in low and inhomogeneous contrast predictions. SCOPE maintained high signal in the full slice ROI and high contrast in the localized kidney optimization. While not a requirement of the optimization, the spatial exclusivity of the modes in SCOPE contributed to improved performance, especially in the local ROI setting when focusing on contrast. Figure 6 shows SAR maps generated from the two SCOPE modes in Figure 5. Similar to the B_1_^+^ maps, the SAR maps are also spatially exclusive and have the peak SAR occurring in different spatial locations, effectively SAR hopping between the two modes to lower the cumulative pSAR across the modes thus supporting the ability to reduce TR.

**FIGURE 7:**
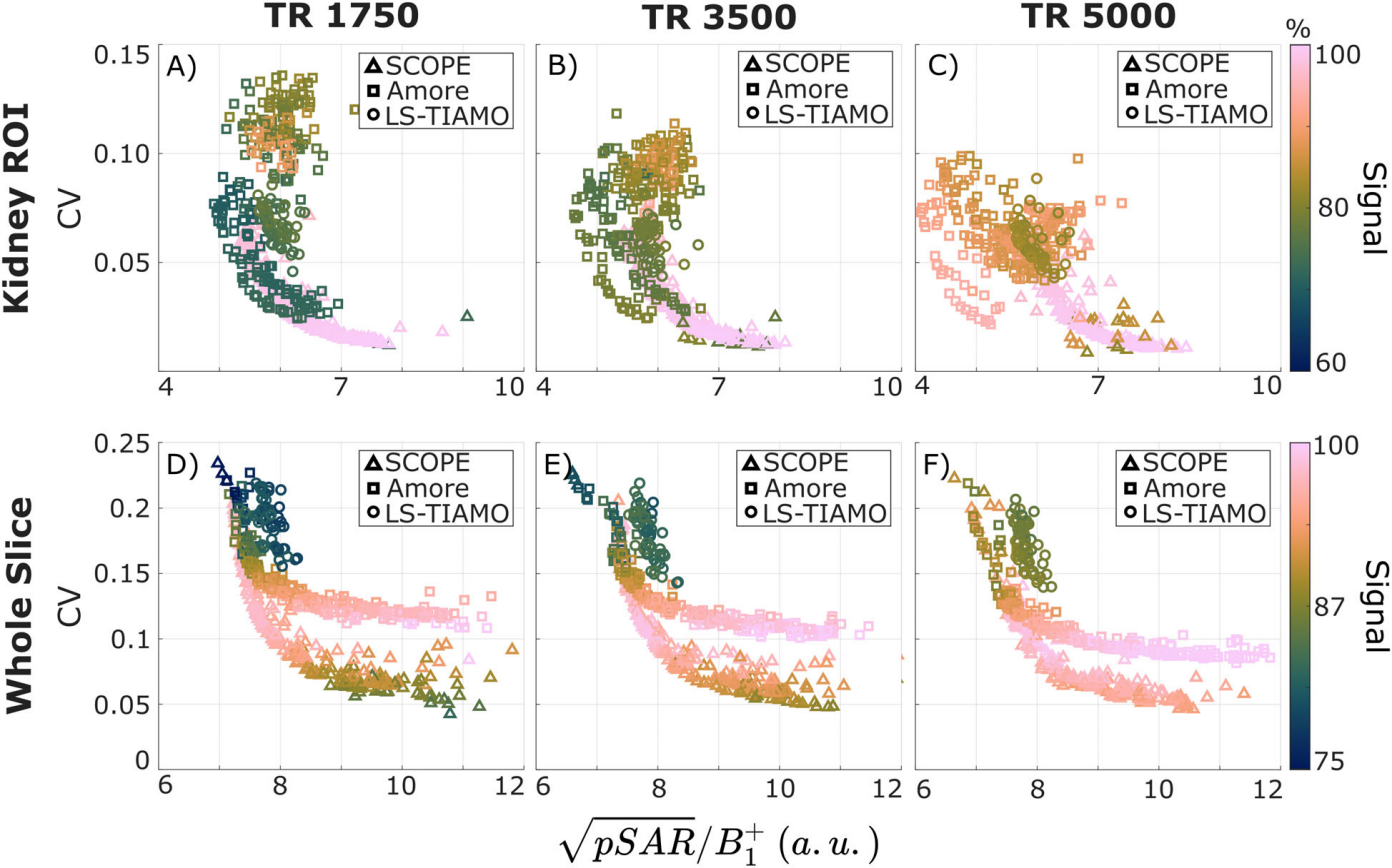
L‐curves calculated by varying the level of CV regularization, zoomed into a region to show typical AMORE and SCOPE performance. All L‐curves had poorly performing LS‐TIAMO solutions which are omitted for clarity but are shown in Figure S2. The top row displays L‐curves calculated using the kidneys as the ROI and the bottom row an entire slice as the ROI. We see that AMORE was able to calculate modes in the kidneys with a better B_1_^+^/sqrt(pSAR) to CV trade‐off compared to SCOPE. However, the SCOPE solutions had higher signal, which would result in higher SNR images. In the bottom row, we see that AMORE and SCOPE find nearly identical shims when optimizing only for pSAR efficiency, but SCOPE is able to reduce CV more than AMORE when higher pSAR is allowed.

L‐curves comparing CV versus normalized pSAR (i.e., pSAR/mean(S)) across a range of homogeneity weights are shown in Figure 7. For each scenario, CV performance is encoded in the *y*‐axis, $\sqrt{\text{pSAR}}/\text{signal}$ efficiency was encoded in the *x*‐axis and mean signal was encoded by varying the color of each point on the curve. For the kidney‐specific ROI, SCOPE and AMORE performed very similarly, although SCOPE did find higher signal solutions. Figure 7a shows LS‐TIAMO solutions that have high CV, low signal, and relatively high pSAR. These solutions originate from the large deviation between the RSOS based signal prediction and the more accurate EPG signal model. However, for some lambda parameters, LS‐TIAMO can find reasonable solutions that produce a high signal, although typically at a higher CV. In the full‐slice L‐curves, SCOPE outperformed the other methods, particularly in the low‐CV regime. We would like to note that AMORE does perform very well in the higher CV regimes using the empirically determined MIP combined with FA_min_ and FA_max_ parameters. These results strongly suggest that the signal‐based optimization in SCOPE enables more consistent and better performance trade‐offs than previous optimization methods.

### 3.4 In Vivo Imaging

**FIGURE 8:**
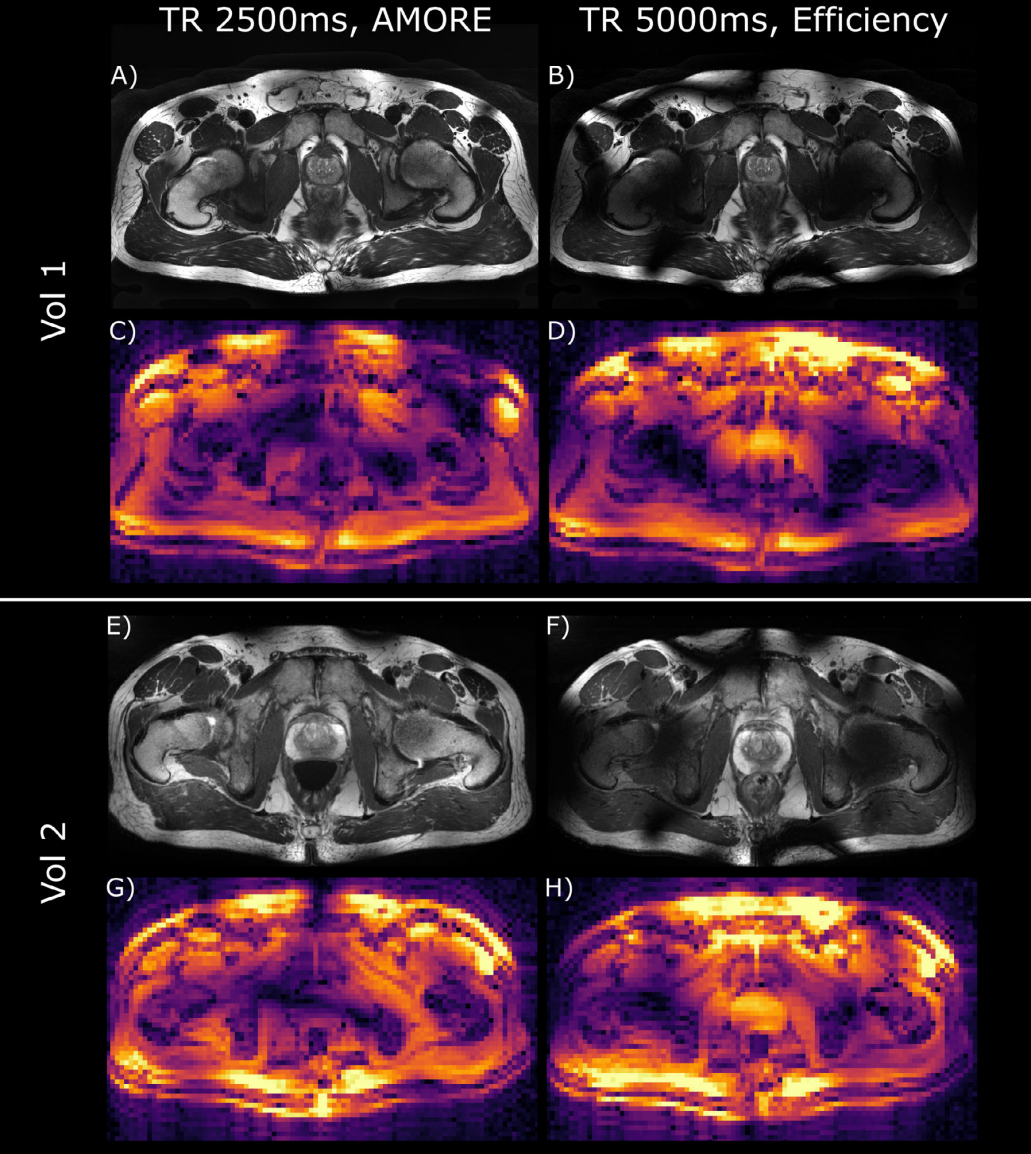
In vivo prostate images acquired with a single shim (B, F) and with two TIAMO modes (A, E) calculated with the AMORE loss function, and the respective B_1_^+^ maps (C, D, G, H). The TR was cut in half for the two TIAMO modes to maintain the same scan time. Even when cutting the TR in half, contrast was maintained in the prostate since AMORE naturally found a complementary second mode that has a B_1_^+^ null band across the prostate (C, G). Note that both the TIAMO and static shimmed images have been bias corrected [31] using the same bias correction map calculated using the TIAMO images.

**FIGURE 9:**
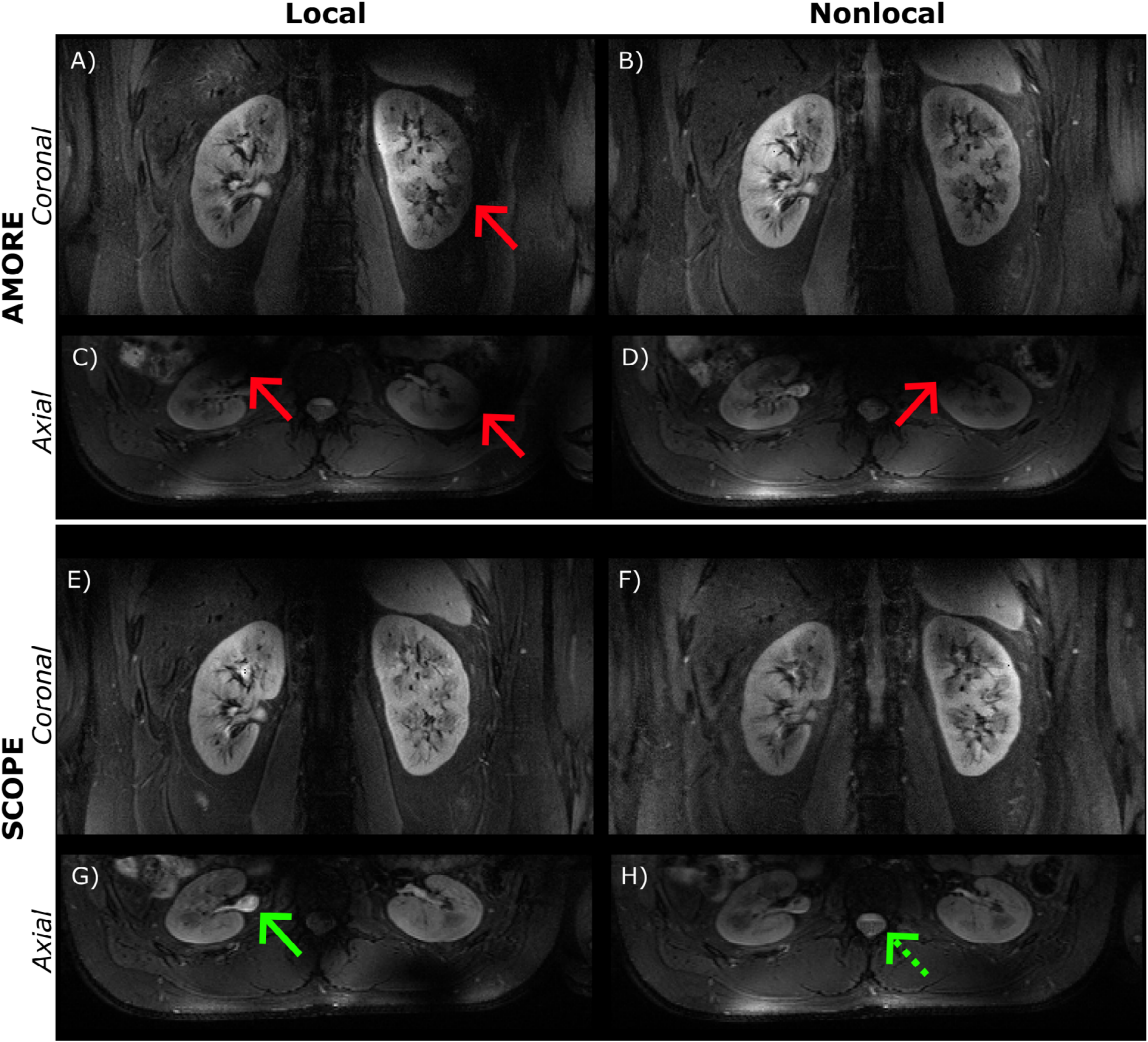
In vivo coronal and axial TSE imaging acquisitions of the kidney to compare multiple AMORE and SCOPE optimization strategies. The localized and non‐localized AMORE solutions provide similar results with modest performance in both the kidney and surrounding anatomy but does have some signal dropout in the kidneys, even with a localized solution (red arrows). The SCOPE solution provides higher SNR and contrast in the kidney with the localized solution (green arrow). Additionally, a more uniform signal throughout the kidneys and surrounding anatomy can be achieved with the non‐localized solution, as appreciated by the contrast in the spinal canal (dashed green arrow).

**FIGURE 10:**
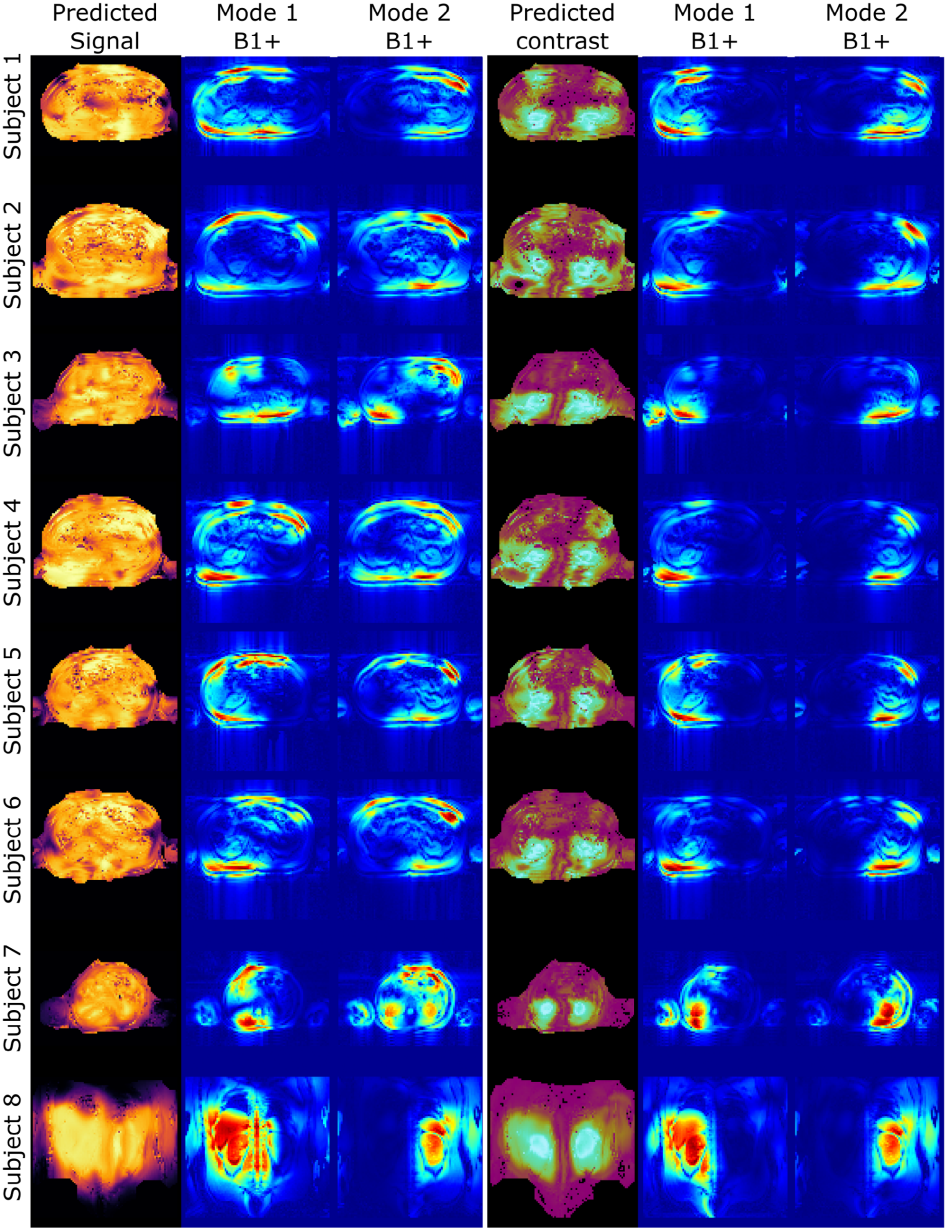
SCOPE shimming results in eight subjects showing whole‐FOV optimization for signal on the (left) and kidney contrast (right). For each volunteer, SCOPE was able to find shims that are predicted to produce good signal and/or good contrast for the regions targeted. All anatomies shown are in the axial orientation except for Subject 8, which is oriented coronally.

Representative in vivo TSE images acquired with SCOPE and AMORE shims are shown in Figures 8, 9, 10. The prostate images shown in Figure 8 demonstrate that in some instances, the AMORE solution finds modes that are spatially exclusive, thereby enabling the halving of TR while still preserving contrast. In larger kidney anatomy and at a more challenging TR of 1750 ms, the AMORE solution does not find spatially exclusive modes, which results in a drop in image quality, as shown in Figure 9. In contrast, SCOPE calculated modes had a high degree of spatial exclusivity, which preserved signal and contrast in the local and non‐local ROIs. The B_1_^+^ maps and signal/contrast prediction maps corresponding to the images in Figure 8 are shown in Figure S3. Retrospective under‐sampling experiments shown in Figure S4 confirm that the virtual channel parallel imaging reconstruction enabled by TIAMO reduced the noise penalty associated with higher accelerations. Figure 10 shows local and non‐local SCOPE shimming results in eight unique volunteers and demonstrates that the SCOPE method is robust to variable body shapes and imaging targets.

## 4 Discussion

This work introduces a new RF shimming framework, SCOPE, designed to address the long‐standing tradeoff between image quality and scan time in TIAMO‐based TSE imaging at UHF. By prescribing a reduced TR equal to half that of a conventional static‐shim protocol, SCOPE eliminates the acquisition time penalty traditionally associated with TIAMO without requiring an increase in parallel imaging acceleration. To maintain contrast under this reduced‐TR regime, we introduced an EPG‐based signal model, pre‐computed a dictionary of signal predictions, and implemented a real‐time shimming pipeline that utilizes the signal dictionary to calculate TIAMO modes. While the framework still requires user‐defined weights to balance competing objectives such as homogeneity and SAR efficiency, these parameters are physically interpretable and directly map to imaging trade‐offs. The result is an optimization strategy that preserves contrast, improves homogeneity, and reduces pSAR compared to previous TIAMO methods.

The performance gains achieved by SCOPE stem from three key mechanisms: accurate signal modeling, spatial mode exclusivity, and flexible trade‐off control. First, by explicitly modeling the TSE TIAMO signal evolution using the new EPG model, SCOPE accounts for the nonlinear dependence of the signal on flip angles from the two modes, the interleaving of excitation modes, and sequence timing. Second, SCOPE naturally finds spatial exclusive modes when optimizing over the new signal model. This ensures that spins experience an effective TR close to the nominal value of the major mode, preserving contrast even when the prescribed TR is halved. It should be noted that the concept of major and minor mode was an implementation decision and SCOPE can just as easily be implemented directly using mode 1 and mode 2 flip angles. Finally, by structuring the optimization as a weighted multi‐objective optimization problem, SCOPE allows users to balance signal efficiency, pSAR, and homogeneity in a transparent and tunable manner. Together, these design features make SCOPE well‐suited to address the demands of large‐FOV TSE imaging at 7 T. While the fine‐tuning of these parameters could be impractical in a clinical setting, universal‐modes [32] based on the new SCOPE model could be investigated.

The development of SCOPE builds directly on the foundational progress made by earlier TIAMO‐based methods, particularly LS‐TIAMO and AMORE. Despite its simplicity, AMORE performs well in particular applications, like prostate TIAMO, and was the inspiration for developing SCOPE and cutting TR in half. At longer TRs, the AMORE modes are more decoupled and longitudinal magnetization is nearly completely restored regardless of the flip angle achieved. However, as the TR is reduced, like those needed in time‐critical applications such as renal breath‐hold imaging, the limitations of the empirical models become more apparent. Furthermore, prior to developing the EPG model, it was unknown how intermediate excitation flip angles would affect contrast. To answer these image contrast questions and developing a more robust TIAMO shimming optimization capable of more directly finding the spatially exclusive modes, we created SCOPE. To quantitatively compare the different TIAMO methods, we ran an L‐curve analysis, plotting homogeneity versus normalized pSAR efficiency across a range of optimization weightings and used marker color to encode mean signal. These curves revealed not only consistent performance improvements with SCOPE, but also important behavioral differences between methods. For example, while AMORE matched SCOPE performance in the high efficiency, high CV regime, it failed to reach the low‐CV regime that SCOPE achieved. In general, LS‐TIAMO exhibited early saturation in signal efficiency as previously reported [16]. Notably, both SCOPE and AMORE produced smooth, monotonic L‐curves across all parameter values and random initializations, which demonstrates the methods' robustness to local minima, a key requirement for reliability, which is critical in a clinical setting. This perspective positions L‐curves not only as a tool for comparing performance but also as a diagnostic indicator of algorithmic stability. An extension of this L‐curve analysis that could be explored in future works, which could be of interest for TSE FA scheduling optimization methods, would be Pareto front manifolds that show optimal performance over both tradeoff parameters pSAR and CV [33].

This study focused on TIAMO acquisitions with two modes but could be extended to include more modes. However, the concept of spatially exclusive modes breaks down when increasing to more than two modes and previous studies have shown that there is only a marginal benefit in going beyond two [32]. The other degree of freedom in generating the signal model is the TSE ETL. While the dictionary was based off an ETL of 9, longer echo trains can be prescribed on the scanner for different imaging scenarios. The effect of variable ETL was examined by creating dictionaries with ETL = 15 and ETL = 23. Combining the variable ETL into a seventh dimension in the signal dictionary and taking an SVD as described in the methods, the dictionary is still close to singular, with the largest singular value more than 20 times the second largest singular value (see Figure S5).

TIAMO is not the only method that has been developed to mitigate the B_1_^+^ inhomogeneity issue at UHF. There are many different RF shimming methods, such as spokes [9, 34, 35], kt‐points [36], SPINS [37], and direct‐signal‐control [8]. However, the importance of accurate calibration scans and the time needed to compute optimal solutions compromises their utilization. SCOPE is a simple method that does not require B_0_ maps, works in the large FA regime needed for TSE imaging, and is capable of producing homogeneous images in a large FOV. A direct competitor to the TIAMO approach is direct signal control (DSC), which also utilizes dynamically applied static shims [8, 18, 38, 39, 40]. The difference between the two methods is that TIAMO changes the RF shims every TR while DSC changes the shim at every refocusing pulse and keeps this set of shims the same for every TR. Ideally, this enables homogeneous imaging without needing to double the number of phase‐encode lines. However, calculating DSC shims can be slow, taking on the order of minutes since they typically need to calculate EPG signals on the fly. A method similar to the precomputed dictionary method presented in this work is possible for DSC but we believe it would be difficult to implement since RF phase across echoes would need to be present in the dictionary. A simple implementation of this would exponentially increase required memory to store the dictionary. To put it in perspective, the current dictionary, when stored in double precision, takes up around 700 MB. Stepping through 10 phase settings for five refocusing pulses would increase this memory demand by 10^5^, resulting in a dictionary that takes up 70 TB. Secondly, DSC does not provide many more degrees of freedom when acquiring PD or T1‐weighted TSE images since most of the signal comes from early echoes in the refocusing train. Furthermore, no previous DSC studies have shown L‐curves, either due to the time to compute them or due to the high likelihood of local minima during the calculation, which would manifest as a large spread of shimming solutions that are not on the Pareto front. Using the SCOPE method, we precompute a dictionary that enables mode calculation in seconds and have shown our optimization strategy is semi‐robust to local minima via the L‐curve plots.

Finally, DSC and TIAMO can be combined by having one TR with one DSC‐based excitation and refocusing train and the next TR could use another unique excitation and refocusing train. Using a combination of these two methods, one might be able to limit the number of unique refocusing shims down to 2 or 3 instead of the previously reported [38] 13, thereby reducing computation time while still maintaining the benefits of both approaches.

Although not explicitly optimized for g‐factor improvements, the highly orthogonal modes found with SCOPE generate virtual channels that are more independent, potentially improving parallel imaging performance compared to previous methods. Future work could explore direct optimization for improved acceleration performance by utilizing more sophisticated reconstruction methods like PRIMO [41], ESPIRiT [42], and TxLR [43, 44].

## 5 Conclusion

We introduced SCOPE, an optimization framework that utilizes a new TSE‐TIAMO signal model to calculate spatially exclusive RF modes for improved signal and contrast at a low TR. By leveraging this EPG signal model, SCOPE formulates a multi‐objective loss function that balances signal efficiency, pSAR, and homogeneity. By precomputing a dictionary, this optimization can operate in real time, taking on the order of seconds to find optima. Furthermore, the implementation of a multi‐objective loss with pSAR and CV terms leads to improved interpretability over contrast and safety trade‐offs. L‐curve evaluations against LS‐TIAMO and AMORE demonstrated SCOPE's superior performance in challenging regimes such as short‐TR kidney imaging and also confirmed its robustness against local minima during optimization. These features make SCOPE a practical and reproducible approach for clinical UHF imaging, with broad applicability across anatomical targets and potential extension to other sequence families.

## Funding

This work was supported by National Institutes of Health (P41 EB027061 and R01 EB029985).

## Supporting information


**FIGURE S1:** Calibration images for LS‐TIAMO and AMORE. LS‐TIAMO predicts signal as a root‐sum‐of‐squares (RSOS) combination of the two modes with a RSOS combination value of 1 being considered power calibrated. We then assign a triangular signal calibration function with zero signal at 0% and 200% calibration and a peak signal at 100% to approximate signal behavior at different power calibrations. AMORE predicts signal as a max‐intensity projection (MIP) and power calibration is assigned by setting minimum and maximum flip angle thresholds, creating a kind of plateau loss function shape. In our implementation, the minimum FA threshold was set to 80% and the maximum to 140% of ideal calibration, similar to what was reported in the original manuscript. Signal prediction inside these FA bounds take a value of 1 and then linearly decreased to 0 at 0% and 200% power calibration.
**FIGURE S2:** Full pSAR/B_1_^+^ by CV space shimming solutions for the different TIAMO methods. While Figure 7 shows a zoomed in version, where the LS‐TIAMO solutions are actually reasonable performance (the darker, slightly higher CV clusters still in the good performance region in these images), there are many LS‐TIAMO solutions that would have very poor imaging performance, shown by the clusters of solutions in the top right of all the plots.
**FIGURE S3:** In vivo kidney images acquired at 7 T with two AMORE‐C modes and a single shim. We can see the signal drop out in the top of the kidney and spine with a single shim and that the virtual channel reconstruction resulted in better signal quality at higher acceleration.
**FIGURE S4:** In vivo Kidney images from Figure 8 showing the output signal prediction maps and B1+ maps for the two modes.
**FIGURE S5:** SVD analysis of the dictionary when compressing ETLs of 9, 15, and 23 into single B1 signal dependent maps and relaxation dependent signal maps. As expected, the dictionary becomes less singular as compared to compression when only considering the ETL = 9 dictionary. However, having a largest singular value nearly 30× larger than the second singular value demonstrates that ETL has little impact on predicted B1 and relaxation dependent signal maps.

**TABLE S1:** Acquisition parameters, note that the relative mapping performed for kidney and liver imaging used only three slices and a 100 ms TR so the acquisition could be performed in a single breath‐hold.

## Data Availability

The data that support the findings of this study are openly available in Data Repository for U of M (DRUM) at https://doi.org/10.13020/zddj‐z486.
