# Supplementary material for: Signal and Contrast Optimization With Predicted Excitations (SCOPE) for Accelerating Large FOV Body Imaging at UHF
Source: Magn Reson Med. 2026 Mar 26;96(2):635–49. doi: 10.1002/mrm.70362 (PMC13269239; doi:10.1002/mrm.70362)
Supplement: Supplementary file 1 — FIGURE S1: Calibration images for LS‐TIAMO and AMORE. LS‐TIAMO predicts signal as a root‐sum‐of‐squares (RSOS) combination of the two modes with a RSOS combination value of 1 being considered power calibrated. We then assign a triangular signal calibration function with zero signal at 0% and 200% calibration and a peak signal at 100% to approximate signal behavior at different power calibrations. AMORE predicts signal as a max‐intensity projection (MIP) and power calibration is assigned by setting minimum and maximum flip angle thresholds, creating a kind of plateau loss function shape. In our implementation, the minimum FA threshold was set to 80% and the maximum to 140% of ideal calibration, similar to what was reported in the original manuscript. Signal prediction inside these FA bounds take a value of 1 and then linearly decreased to 0 at 0% and 200% power calibration. FIGURE S2: Full pSAR/B1 + by CV space shimming solutions for the different TIAMO methods. While Figure 7 shows a zoomed in version, where the LS‐TIAMO solutions are actually reasonable performance (the darker, slightly higher CV clusters still in the good performance region in these images), there are many LS‐TIAMO solutions that would have very poor imaging performance, shown by the clusters of solutions in the top right of all the plots. FIGURE S3: In vivo kidney images acquired at 7 T with two AMORE‐C modes and a single shim. We can see the signal drop out in the top of the kidney and spine with a single shim and that the virtual channel reconstruction resulted in better signal quality at higher acceleration. FIGURE S4: In vivo Kidney images from Figure 8 showing the output signal prediction maps and B1+ maps for the two modes. FIGURE S5: SVD analysis of the dictionary when compressing ETLs of 9, 15, and 23 into single B1 signal dependent maps and relaxation dependent signal maps. As expected, the dictionary becomes less singular as compared to compression when only considering the ETL = 9 dict [file MRM-96-635-s001.docx]

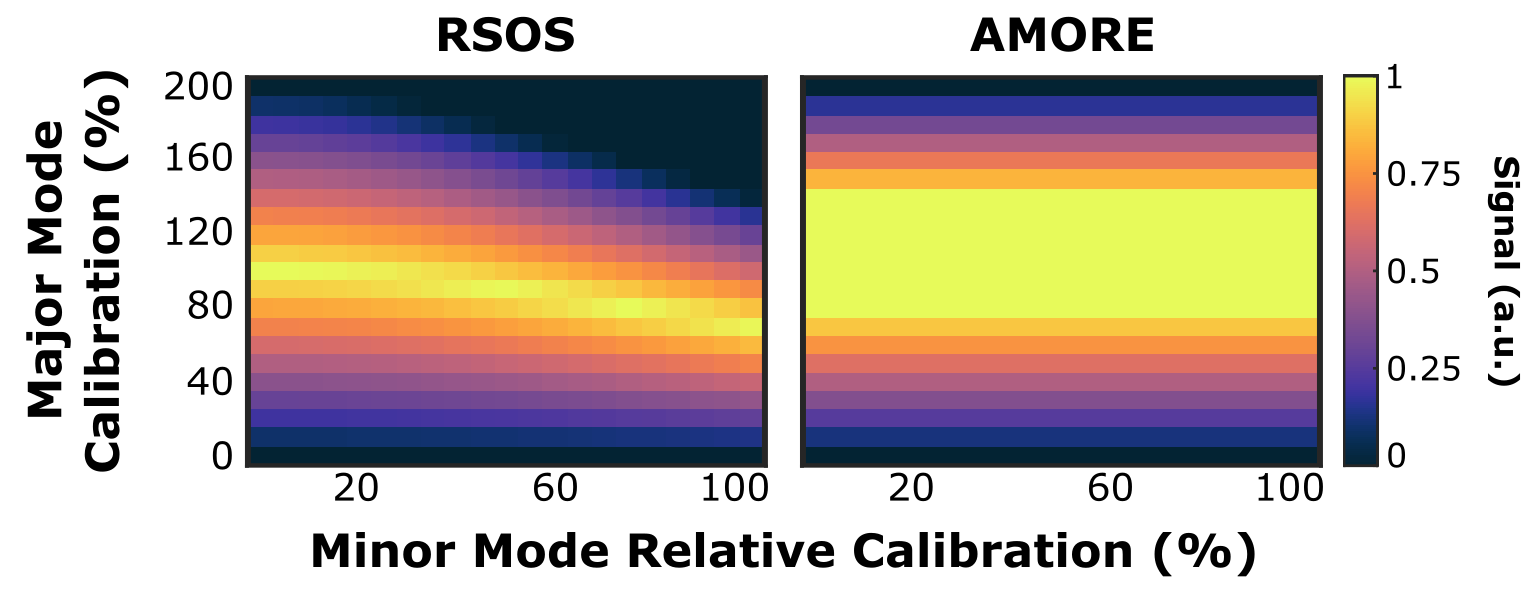


**Figure S1:** Calibration images for LS-TIAMO and AMORE. LS-TIAMO predicts signal as a root-sum-of-squares (RSOS) combination of the two modes with a RSOS combination value of 1 being considered power calibrated. We then assign a triangular signal calibration function with zero signal at 0% and 200% calibration and a peak signal at 100% to approximate signal behavior at different power calibrations. AMORE predicts signal as a max-intensity projection (MIP) and power calibration is assigned by setting minimum and maximum flip angle thresholds, creating a kind of plateau loss function shape. In our implementation, the minimum FA threshold was set to 80% and the maximum to 140% of ideal calibration, similar to what was reported in the original manuscript. Signal prediction inside these FA bounds take a value of 1 and then linearly decreased to 0 at 0% and 200% power calibration.


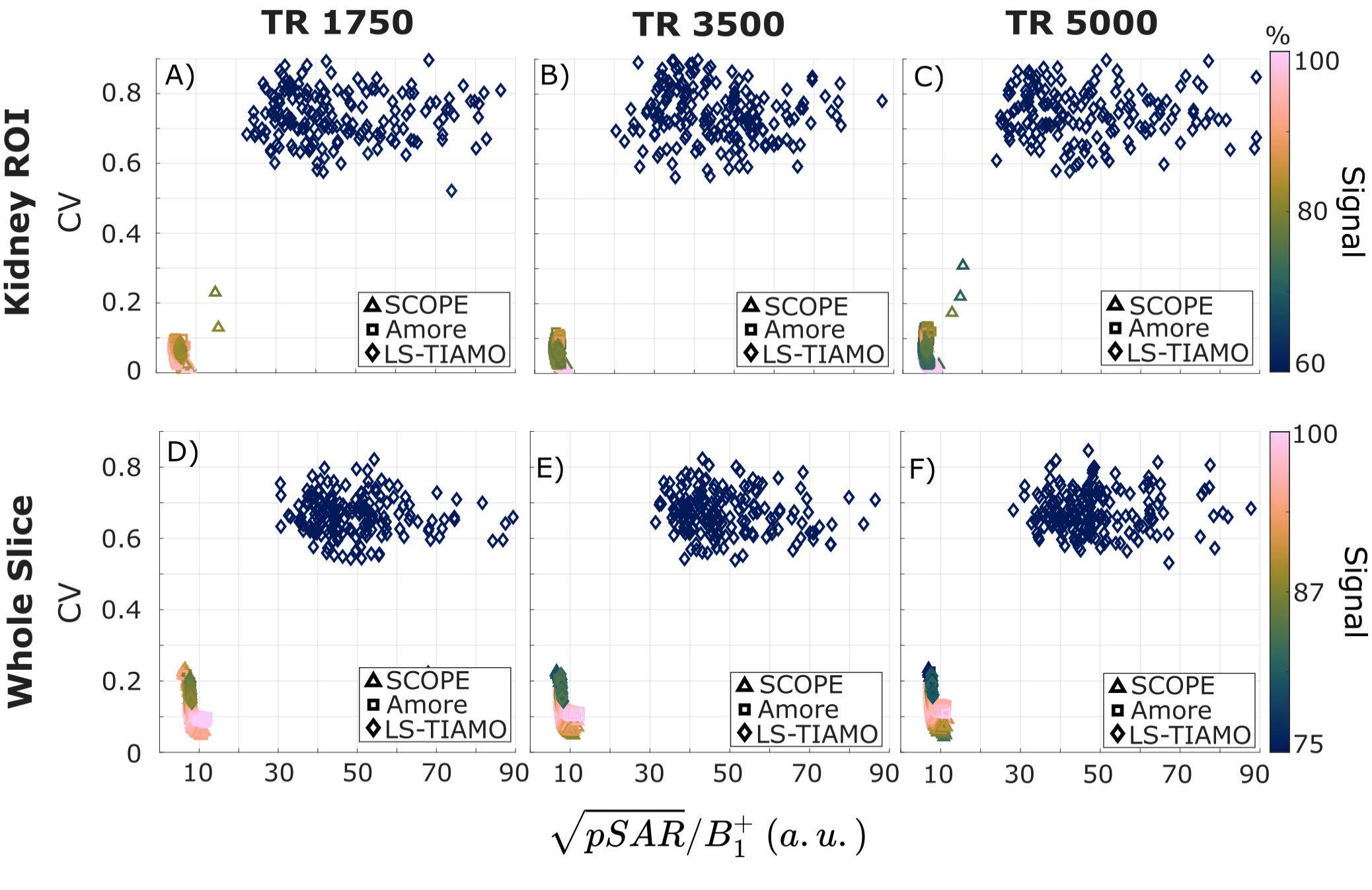


**Figure S2:** Full pSAR/B_1_^+^ by CV space shimming solutions for the different TIAMO methods. While figure 7 shows a zoomed in version, where the LS-TIAMO solutions are actually reasonable performance (the darker, slightly higher CV clusters still in the good performance region in these images), there are many LS-TIAMO solutions that would have very poor imaging performance, shown by the clusters of solutions in the top right of all the plots.


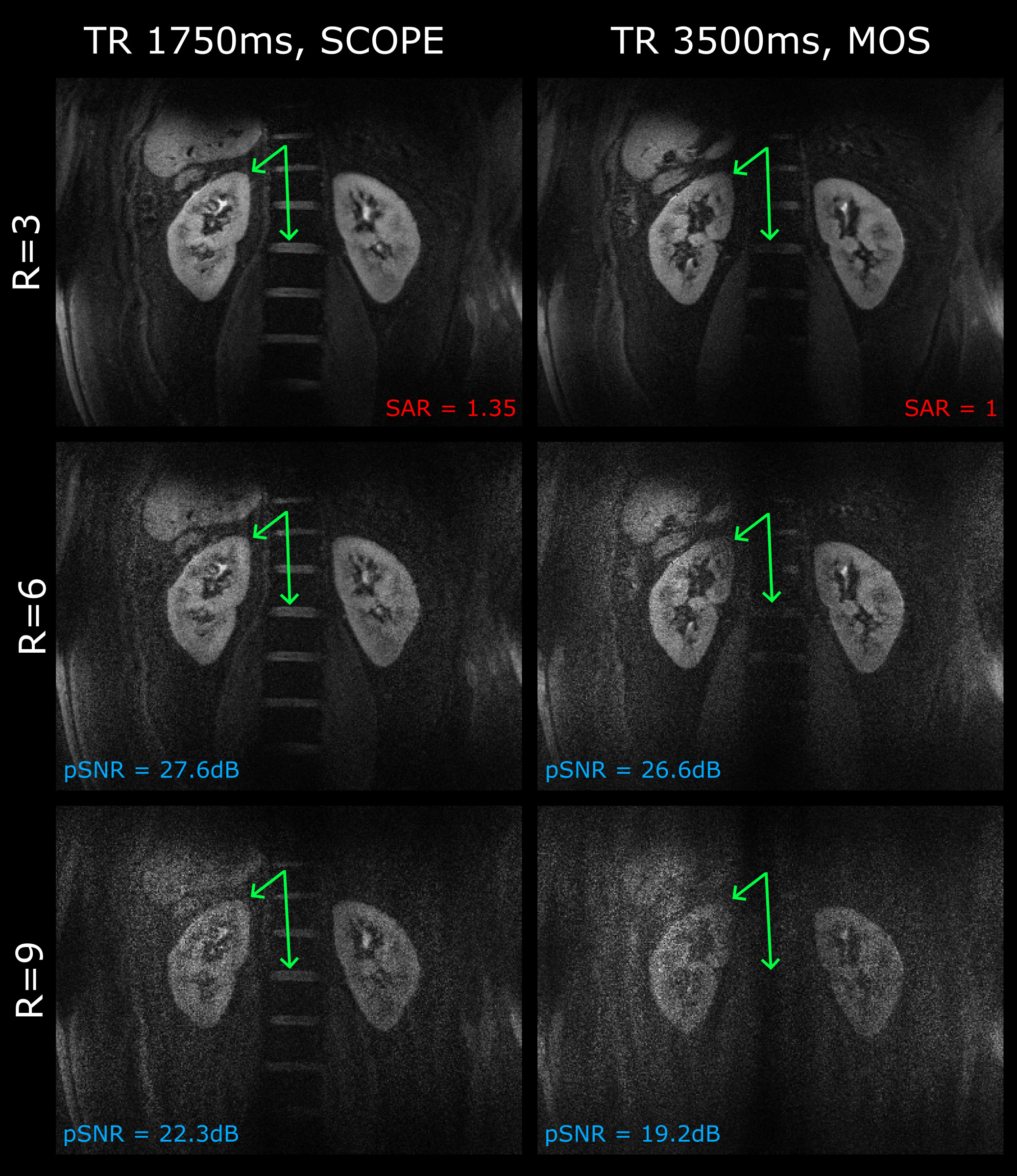


**Figure S3:** In-vivo kidney images acquired at 7T with two AMORE-C modes and a single shim. We can see the signal drop out in the top of the kidney and spine with a single shim and that the virtual channel reconstruction resulted in better signal quality at higher acceleration.


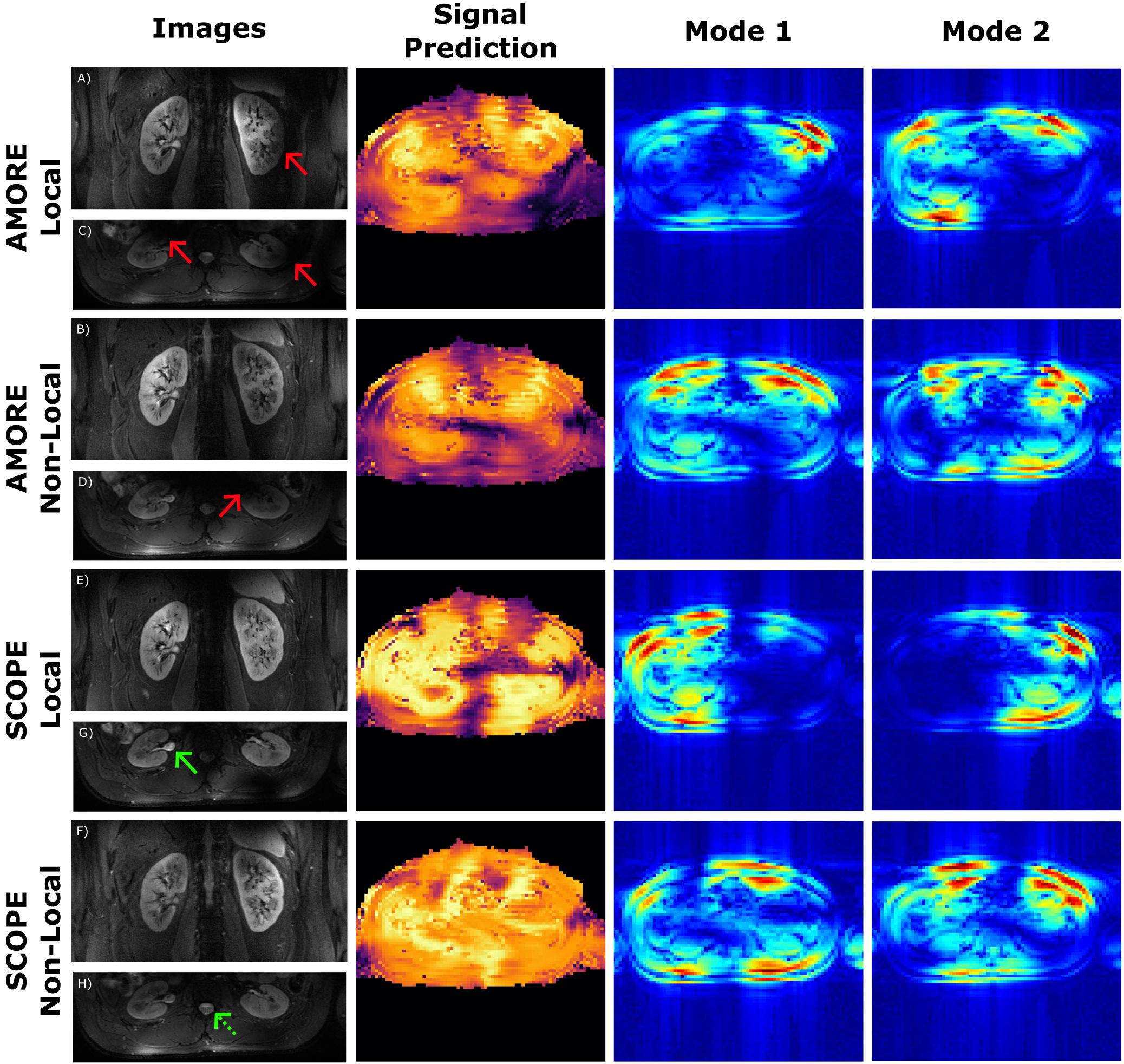


**Figure S4:** In-vivo Kidney images from figure 8 showing the output signal prediction maps and B1+ maps for the two modes


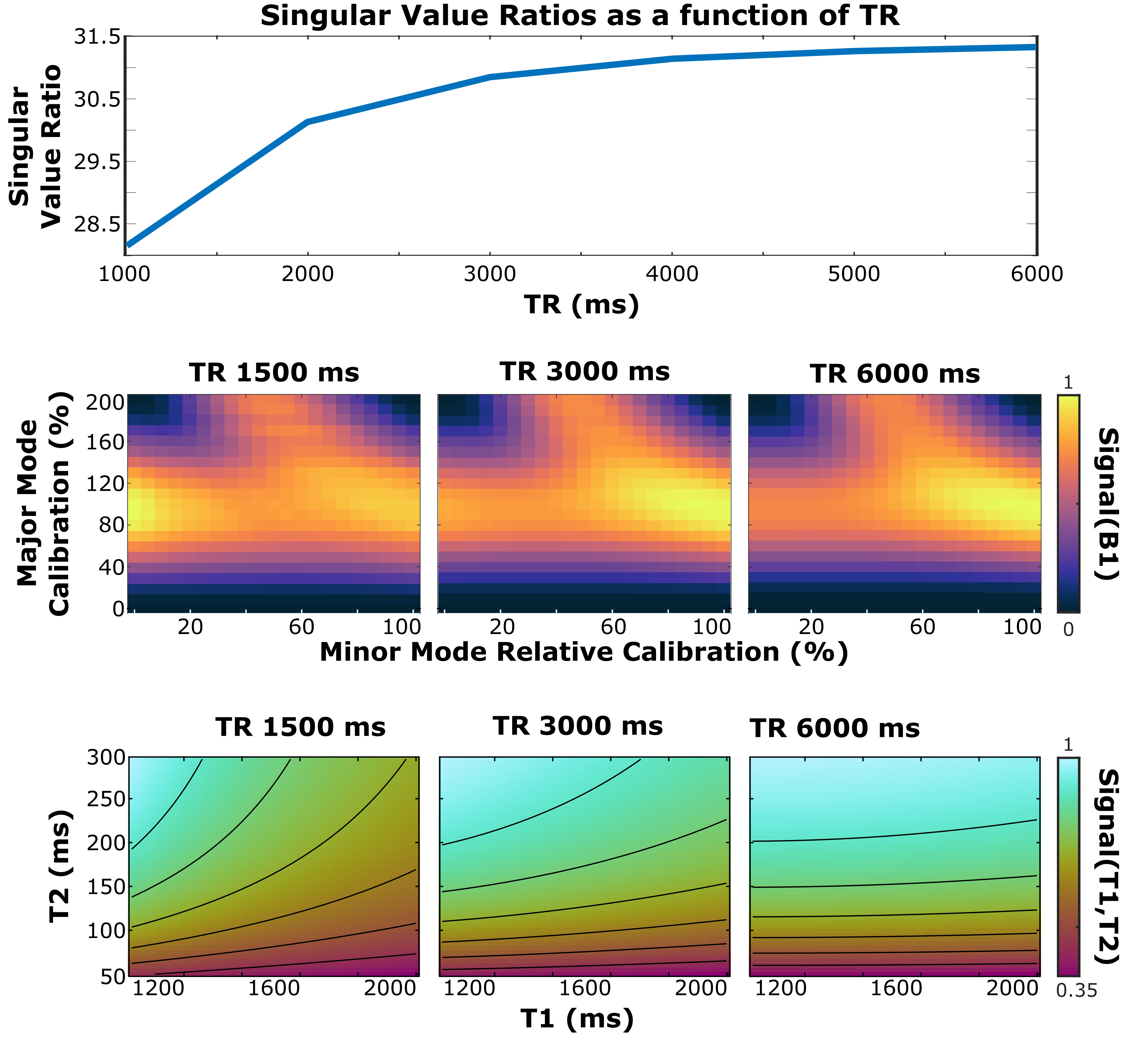


**Figure S5:** SVD analysis of the dictionary when compressing ETLs of 9, 15, and 23 into single B1 signal dependent maps and relaxation dependent signal maps. As expected, the dictionary becomes less singular as compared to compression when only considering the ETL=9 dictionary. However, having a largest singular value nearly 30x larger than the second singular value demonstrates that ETL has little impact on predicted B1 and relaxation dependent signal maps.
