# Supplementary material for: Signal and Contrast Optimization With Predicted Excitations (SCOPE) for Accelerating Large FOV Body Imaging at UHF
Source: Magn Reson Med. 2026 Mar 26;96(2):635–49. doi: 10.1002/mrm.70362 (PMC13269239; doi:10.1002/mrm.70362)
Supplement: Supplementary file 2 — TABLE S1: Acquisition parameters, note that the relative mapping performed for kidney and liver imaging used only three slices and a 100 ms TR so the acquisition could be performed in a single breath‐hold. [file MRM-96-635-s002.docx]

|  | Relative B­_1_^+^ mapping | AFI | TFL | Kidney | Kidney | Prostate |
| --- | --- | --- | --- | --- | --- | --- |
| Orientation | Transverse | Transverse | Transverse | Transverse | Coronal | Transverse |
| Fat Sat | No | No | No | Yes | Yes | No |
| TR (ms) | 64 | 115/15 | 1230 | 1750 | 1750 | 3000 |
| TE (ms) | 3.06 | 3.06 | 1.84 | 71 | 71 | 65 |
| FA (°) | 15 | 40 | 80 | 120 | 120 | 140 |
| FOV (mm^2^) | 475 x 260 | 475 x 475 | 450x450 | 388x300 | 375 X 280 | 375 x 250 |
| Slices | Variable | 18 | 3 | 5 | 5 | 15 |
| Resolution (mm^3^) | 4 x 4 x 5 | 3.7 x 3.7 x 5 | 4 x 4 x 5 | 1 x 1 x 3 | 1 x 1 x 3 | 0.75 x 0.4 x 3 |
| R | 1 | 3 | 1 | 3 | 3 | 2 |
| ETL | - | - | - | 21 | 21 | 9 |
| BW (Hz/Px) | 600 | 490 | 300 | 400 | 217 | 405 |
| TA (min:sec) | variable | 2:19 | 0:04 | 0:28 | 0:28 | 3:30 |
|  |  |  |  |  |  |  |

TABLE S1: Acquisition parameters, note that the relative mapping performed for kidney and liver imaging used only 3 slices and a 100ms TR so the acquisition could be performed in a single breath-hold.

Abbreviations: flip angle (FA), fat suppressed (FS), actual flip angle imaging (AFI), Turbo FLAHS (TFL), parallel imaging reduction/acceleration factor (R), field of view (FOV), echo train length (ETL), readout bandwidth (BW), time of acquisition (TA), signal to noise ratio (SNR).
